# Supplementary material for: Bone Marrow Aspirate Concentrate versus Human Umbilical Cord Blood-Derived Mesenchymal Stem Cells for Combined Cartilage Regeneration Procedure in Patients Undergoing High Tibial Osteotomy: A Systematic Review and Meta-Analysis
Source: Medicina (Kaunas). 2023 Mar 22;59(3):634. doi: 10.3390/medicina59030634 (PMC10059261; doi:10.3390/medicina59030634)
Supplement: Supplementary file 1 [file medicina-59-00634-s001.zip › Supplemental Table S1.pdf]

Table S1. Keywords and search details.

DB	Search	Keywords / Search details
**PubMed**	#1	"Osteoarthritis, Knee"[Mesh]
	#2	"Osteoarthritis, Knee"[TW] OR "Knee Osteoarthritides"[TW] OR "Knee Osteoarthritis"[TW] OR "Osteoarthritis of Knee"[TW] OR "Osteoarthritis of the Knee"[TW] OR "knee arthritis"[TW] OR "arthrosis, knee"[TW] OR "femorotibial arthrosis"[TW] OR "gonarthrosis"[TW] OR "knee arthrosis"[TW] OR "knee joint arthrosis"[TW] OR "knee joint osteoarthritis"[TW] OR "knee osteo-arthritis"[TW] OR "knee osteo-arthrosis"[TW] OR "knee osteoarthrosis"[TW] OR "osteoarthrosis, knee"[TW]
	#3	"osteotomy"[Mesh] OR "osteotomy"[TW] OR "Osteotomies"[TW] OR "corrective osteotomy"[TW]
	#4	"high tibial osteotomy"[TW] OR "high (proximal) tibial osteotomy"[TW] OR "high tibia osteotomy"[TW] OR "high tibia osteotomy"[TW] OR "high tibial osteotomy"[TW] OR "high tibial osteotomy procedure"[TW] OR "high tibial osteotomy surgery"[TW] OR "high tibial osteotomy techniques"[TW] OR "HTO procedure"[TW] OR "HTO surgery"[TW] OR "HTO technique"[TW] OR "proximal tibia osteotomy"[TW] OR "proximal tibial osteotomy"[TW] OR "tibia proximal corrective osteotomy"[TW] OR "tibia proximal osteotomy"[TW] OR "tibial proximal osteotomy"[TW] OR "HTO"[TW]
	**#5**	**(#1 OR #2) AND (#3 OR #4)**
	#6	"Umbilical Cord"[Mesh] OR "Umbilical Cord"[TW] OR "Umbilical Cords"[TW]
	#7	"Mesenchymal Stem Cells"[Mesh] OR "Mesenchymal Stem Cells"[TW] OR "Mesenchymal Stem Cell"[TW]
	#8	"UC-mesenchymal stem cells"[TW] OR "UC-MSC (umbilical cord mesenchymal stem cell)"[TW] OR "umbilical cord (UC) -mesenchymal stem cells (MSC)"[TW] OR "umbilical cord mesenchymal stem cells"[TW] OR "umbilical cord MSC"[TW] OR "umbilical cord MSC (UC-MSC)"[TW] OR "umbilical cord-derived mesenchymal stem cell"[TW] OR "umbilical cord-derived mesenchymal stem cells"[TW]
	#9	"umbilical cord blood-derived mesenchymal stem cell"[TW] OR "UCB-MSC"[TW] OR "hUCB-MSC"[TW]

	#10	"bone marrow aspirate concentrate"[TW] OR "bone marrow aspirate"[TW] OR "BMAC"[TW] OR "bone marrow aspiration concentrate"[TW] OR "bone marrow aspiration"[TW] OR (bone[TW] AND marrow[TW] AND aspirate[TW]) OR (("bone marrow"[Mesh] OR "bone marrow"[TW]) AND ("aspirate"[TW] OR "concentrate"[TW]))
	**#11**	**(#6 AND #7) OR #8 OR #9 OR #10**
	**#12**	**#5 AND #11**

DB	Search	Keywords / Search details
**EMBASE**	#1	"knee osteoarthritis"/exp
	#2	"Osteoarthritis, Knee":ti,ab,kw,de OR "Knee Osteoarthritides":ti,ab,kw,de OR "Knee Osteoarthritis":ti,ab,kw,de OR "Osteoarthritis of Knee":ti,ab,kw,de OR "Osteoarthritis of the Knee":ti,ab,kw,de OR "knee arthritis":ti,ab,kw,de OR "arthrosis, knee":ti,ab,kw,de OR "femorotibial arthrosis":ti,ab,kw,de OR "gonarthrosis":ti,ab,kw,de OR "knee arthrosis":ti,ab,kw,de OR "knee joint arthrosis":ti,ab,kw,de OR "knee joint osteoarthritis":ti,ab,kw,de OR "knee osteo-arthritis":ti,ab,kw,de OR "knee osteo-arthrosis":ti,ab,kw,de OR "knee osteoarthrosis":ti,ab,kw,de OR "osteoarthrosis, knee":ti,ab,kw,de
	#3	"osteotomy"/exp OR "osteotomy":ti,ab,kw,de OR "Osteotomies":ti,ab,kw,de OR "corrective osteotomy":ti,ab,kw,de

	#4	"high tibial osteotomy":ti,ab,kw,de OR "high (proximal) tibial osteotomy":ti,ab,kw,de OR "high tibia osteotomy":ti,ab,kw,de OR "high tibia osteotomy":ti,ab,kw,de OR "high tibial osteotomy":ti,ab,kw,de OR "high tibial osteotomy procedure":ti,ab,kw,de OR "high tibial osteotomy surgery":ti,ab,kw,de OR "high tibial osteotomy techniques":ti,ab,kw,de OR "HTO procedure":ti,ab,kw,de OR "HTO surgery":ti,ab,kw,de OR "HTO technique":ti,ab,kw,de OR "proximal tibia osteotomy":ti,ab,kw,de OR "proximal tibial osteotomy":ti,ab,kw,de OR "tibia proximal corrective osteotomy":ti,ab,kw,de OR "tibia proximal osteotomy":ti,ab,kw,de OR "tibial proximal osteotomy":ti,ab,kw,de OR "HTO":ti,ab,kw,de
	**#5**	**(#1 OR #2) AND (#3 OR #4)**
	#6	"umbilical cord"/exp OR "Umbilical Cord":ti,ab,kw,de OR "Umbilical Cords":ti,ab,kw,de
	#7	"mesenchymal stem cell"/exp OR "Mesenchymal Stem Cells":ti,ab,kw,de OR "Mesenchymal Stem Cell":ti,ab,kw,de
	#8	"UC-mesenchymal stem cells":ti,ab,kw,de OR "UC-MSC (umbilical cord mesenchymal stem cell)":ti,ab,kw,de OR "umbilical cord (UC) -mesenchymal stem cells (MSC)":ti,ab,kw,de OR "umbilical cord mesenchymal stem cells":ti,ab,kw,de OR "umbilical cord MSC":ti,ab,kw,de OR "umbilical cord MSC (UC-MSC)":ti,ab,kw,de OR "umbilical cord-derived mesenchymal stem cell":ti,ab,kw,de OR "umbilical cord-derived mesenchymal stem cells":ti,ab,kw,de
	#9	"umbilical cord blood-derived mesenchymal stem cell":ti,ab,kw,de OR "UCB-MSC":ti,ab,kw,de OR "hUCB-MSC":ti,ab,kw,de
	#10	"bone marrow aspirate concentrate":ti,ab,kw,de OR "bone marrow aspirate":ti,ab,kw,de OR "BMAC":ti,ab,kw,de OR "bone marrow aspiration concentrate":ti,ab,kw,de OR "bone marrow aspiration":ti,ab,kw,de OR (bone:ti,ab,kw,de AND marrow:ti,ab,kw,de AND aspirate:ti,ab,kw,de) OR (("bone marrow"/exp OR "bone marrow":ti,ab,kw,de) AND ("aspirate":ti,ab,kw,de OR "concentrate":ti,ab,kw,de))
	**#11**	**(#6 AND #7) OR #8 OR #9 OR #10**
	**#12**	**#5 AND #11**

DB	Search	Keywords / Search details
**Cochrane Library**	#1	[mh "Osteoarthritis, Knee"]
	#2	"Osteoarthritis, Knee":ti,ab,kw OR "Knee Osteoarthritides":ti,ab,kw OR "Knee Osteoarthritis":ti,ab,kw OR "Osteoarthritis of Knee":ti,ab,kw OR "Osteoarthritis of the Knee":ti,ab,kw OR "knee arthritis":ti,ab,kw OR "arthrosis, knee":ti,ab,kw OR "femorotibial arthrosis":ti,ab,kw OR "gonarthrosis":ti,ab,kw OR "knee arthrosis":ti,ab,kw OR "knee joint arthrosis":ti,ab,kw OR "knee joint osteoarthritis":ti,ab,kw OR "knee osteo-arthritis":ti,ab,kw OR "knee osteo-arthrosis":ti,ab,kw OR "knee osteoarthrosis":ti,ab,kw OR "osteoarthrosis, knee":ti,ab,kw
	#3	[mh "osteotomy"] OR "osteotomy":ti,ab,kw OR "Osteotomies":ti,ab,kw OR "corrective osteotomy":ti,ab,kw
	#4	"high tibial osteotomy":ti,ab,kw OR "high (proximal) tibial osteotomy":ti,ab,kw OR "high tibia osteotomy":ti,ab,kw OR "high tibia osteotomy":ti,ab,kw OR "high tibial osteotomy":ti,ab,kw OR "high tibial osteotomy procedure":ti,ab,kw OR "high tibial osteotomy surgery":ti,ab,kw OR "high tibial osteotomy techniques":ti,ab,kw OR "HTO procedure":ti,ab,kw OR "HTO surgery":ti,ab,kw OR "HTO technique":ti,ab,kw OR "proximal tibia osteotomy":ti,ab,kw OR "proximal tibial osteotomy":ti,ab,kw OR "tibia proximal corrective osteotomy":ti,ab,kw OR "tibia proximal osteotomy":ti,ab,kw OR "tibial proximal osteotomy":ti,ab,kw OR "HTO":ti,ab,kw
	**#5**	**(#1 OR #2) AND (#3 OR #4)**
	#6	[mh "Umbilical Cord"] OR "Umbilical Cord":ti,ab,kw OR "Umbilical Cords":ti,ab,kw
	#7	[mh "Mesenchymal Stem Cells"] OR "Mesenchymal Stem Cells":ti,ab,kw OR "Mesenchymal Stem Cell":ti,ab,kw

	#8	"UC-mesenchymal stem cells":ti,ab,kw OR "UC-MSC (umbilical cord mesenchymal stem cell)":ti,ab,kw OR "umbilical cord (UC) -mesenchymal stem cells (MSC)":ti,ab,kw OR "umbilical cord mesenchymal stem cells":ti,ab,kw OR "umbilical cord MSC":ti,ab,kw OR "umbilical cord MSC (UC-MSC)":ti,ab,kw OR "umbilical cord-derived mesenchymal stem cell":ti,ab,kw OR "umbilical cord-derived mesenchymal stem cells":ti,ab,kw
	#9	"umbilical cord blood-derived mesenchymal stem cell":ti,ab,kw OR "UCB-MSC":ti,ab,kw OR "hUCB-MSC":ti,ab,kw
	#10	"bone marrow aspirate concentrate":ti,ab,kw OR "bone marrow aspirate":ti,ab,kw OR "BMAC":ti,ab,kw OR "bone marrow aspiration concentrate":ti,ab,kw OR "bone marrow aspiration":ti,ab,kw OR (bone:ti,ab,kw AND marrow:ti,ab,kw AND aspirate:ti,ab,kw) OR (([mh "bone marrow"] OR "bone marrow":ti,ab,kw) AND ("aspirate":ti,ab,kw OR "concentrate":ti,ab,kw))
	**#11**	**(#6 AND #7) OR #8 OR #9 OR #10**
	**#12**	**#5 AND #11**
