# Supplementary material for: Bone Marrow Aspirate Concentrate versus Human Umbilical Cord Blood-Derived Mesenchymal Stem Cells for Combined Cartilage Regeneration Procedure in Patients Undergoing High Tibial Osteotomy: A Systematic Review and Meta-Analysis
Source: Medicina (Kaunas). 2023 Mar 22;59(3):634. doi: 10.3390/medicina59030634 (PMC10059261; doi:10.3390/medicina59030634)
Supplement: Supplementary file 1 [file medicina-59-00634-s001.zip › Supplemental Table S2.pdf]

Table S2. Risk-of-bias assessment performed using the MINORS score.

	**Study**	Jin, 2021	Lee, 2021	Yang, 2022	Cavallo, 2018	Song, 2020	Song, 2020b	Chung,2021
	**Level of evidence**	III	III	III	IV	IV	IV	IV
**1**	A clearly stated aim	2	2	2	2	2	2	2
**2**	Inclusion of consecutive patients	2	1	1	0	1	1	2
**3**	Prospective collection of data	0	0	0	1	1	1	1
**4**	Endpoints appropriate to the aim of the study	2	2	2	2	2	2	2
**5**	Unbiased assessment of the study endpoint	1	0	0	0	1	1	1
**6**	Follow-up period appropriate to the aim of the study	1	1	1	1	1	1	1
**7**	Loss to follow up less than 5%	1	2	1	0	1	2	2
**8**	Prospective calculation of the study size	2	0	0	0	0	0	0
**9**	An adequate control group	2	0	0	-	-	-	-
**10**	Contemporary groups	2	2	2	-	-	-	-
**11**	Baseline equivalence of groups	1	1	2	-	-	-	-
**12**	Adequate statistical analyses	2	2	2	-	-	-	-
	**Total score**	**18**	**13**	**13**	**6**	**9**	**10**	**11**

MINORS, Methodological index for non-randomized studies
