# Supplementary figures and images for: Bone Marrow Aspirate Concentrate versus Human Umbilical Cord Blood-Derived Mesenchymal Stem Cells for Combined Cartilage Regeneration Procedure in Patients Undergoing High Tibial Osteotomy: A Systematic Review and Meta-Analysis
Source: Medicina (Kaunas). 2023 Mar 22;59(3):634. doi: 10.3390/medicina59030634 (PMC10059261; doi:10.3390/medicina59030634)

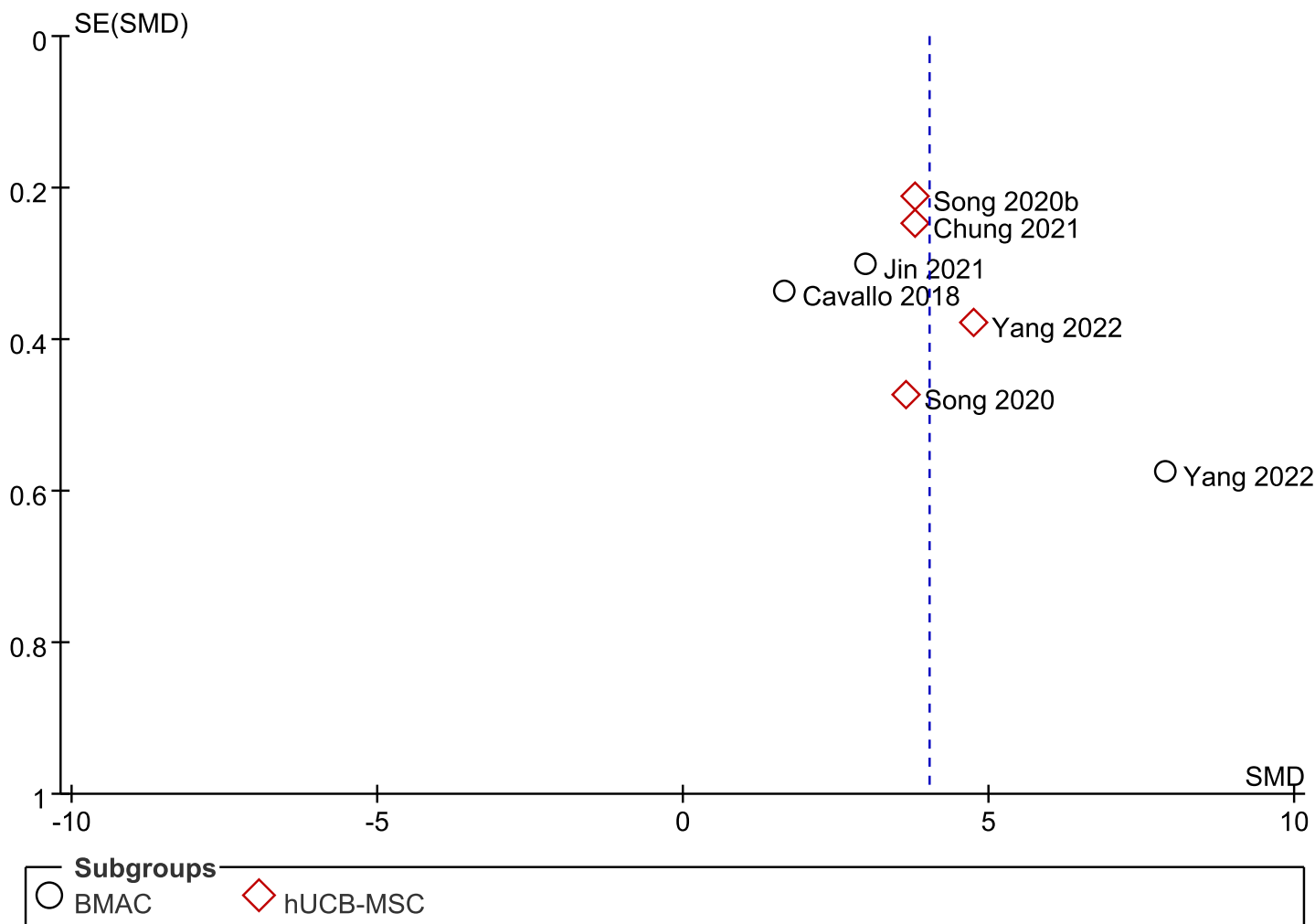

Supplement: Supplementary file 1 [file medicina-59-00634-s001.zip › supplemental Figure S1.pdf]
